# A System of Practical Surgery

**Published:** 1843-04-15

**Authors:** 


*A System of Practical Surgery.* By William Fergusson, F. R. S. E., Professor of Surgery in King's College, London, etc. etc. With two hundred and forty-six Illustrations, from drawings by Baggs, engraved by Gilbert. With Notes and Additional Illustrations. By George W. Norris, M. D., Surgeon to the Pennsylvania Hospital. Philadelphia, 1843. 8vo. pp. 629. Lea & Blanchard.

It is with unfeigned satisfaction that we call the attention of the profession in this country to this excellent work, which, although presenting some of the defects of most of the recent transatlantic publications on the same subject, yet richly deserves the reputation conceded to it, of being the *best practical surgery extant*, at least in the English language.

The matter added by the American editor is both interesting and useful, and bears the impress of his cautious and discriminating mind. The illustrations are also admirably executed, and will contribute much to the reputation of Mr. Gilbert, already known as one of the best of our "workers in wood;" and we unhesitatingly declare, that no American book of this class will compare with it in all the minor details of "getting up." Too much praise cannot be awarded to the enterprising Publishers, to whom the profession is already so largely indebted; and we sincerely trust that their effort to bring out medical works in this style, will meet with the encouragement it so richly deserves. T. D. M.

